# USING DISCURSIVE PSYCHOLOGY TO EXAMINE PATIENT MEDICAL EDUCATION

**DOI:** 10.1093/geroni/igae098.0992

**Published:** 2024-12-31

**Authors:** Sean Halpin

**Affiliations:** RTI International, Atlanta, Georgia, United States

## Abstract

Patient-centered care is facilitated by succinct medical information delivered by nurse patient-educators. However, navigating the array of treatment options poses challenges, especially for patients with complex and co-morbid conditions, such as older adults with multiple myeloma. In the current study, I applied discursive psychology to analyze speech patterns in 12 education visits (1011 minutes of audio) conducted by three nurses. Patients had a median age of 65 (IQR- 54-75), were mostly white (75%), and split between male and female. I identified two overarching speech strategies used including (1) verbatim repeated (unwritten) sentences which nurses piloted and evolved from patient to patient and (2) application of language such as “I” to show authority versus “we” to demonstrate alignment with members of the medical team. Ultimately, nurses used these speech patterns to help recall information, revise their internal scripts, and assure patients.

